# Peer Attachment and Prosocial Behavior: The Mediating Role of Positive Legal Emotion and the Moderating Effect of Social Exclusion

**DOI:** 10.3390/bs16040494

**Published:** 2026-03-26

**Authors:** Weiwei Sun, Shuhui Xu

**Affiliations:** 1 School of Education, Wenzhou University, Wenzhou 325035, China; sunweiwei@wzu.edu.cn; 2 Wenzhou Research Center for Family Tradition and Family Education, Wenzhou 325035, China

**Keywords:** peer attachment, positive legal emotion, prosocial behavior, social exclusion, legal socialization, moderated mediation

## Abstract

Prosocial behavior supports social cohesion and legal order. Drawing on attachment theory and emotional socialization theory, this study examined whether peer attachment promotes prosocial behavior through positive legal emotion, defined as affective identification with and respect for law, and whether social exclusion moderates this pathway. A cross-sectional survey was conducted with 401 Chinese university students. Measures assessed peer attachment, positive legal emotion, social exclusion, and prosocial behavior. Gender and parental education were included as control variables. Correlational and regression analyses were conducted, followed by conditional process modeling using Hayes’ PROCESS Model 60 with 5000 bootstrap resamples to test mediation, moderation, and moderated mediation effects. Peer attachment significantly and positively predicted prosocial behavior. Positive legal emotion partially mediated the relationship between peer attachment and prosocial behavior. Social exclusion significantly moderated the association between peer attachment and positive legal emotion. The indirect effect of peer attachment on prosocial behavior through positive legal emotion became stronger as social exclusion increased. Moderated mediation analyses further confirmed that the indirect effect intensified at higher levels of social exclusion. Positive legal emotion represents an important psychological mechanism linking peer relationships to prosocial outcomes, while social exclusion functions as a key contextual boundary condition. Interventions that strengthen peer support, enhance legal emotional engagement, and reduce social exclusion may promote prosocial and legal socialization among university students and contribute to broader social integration and stability.

## 1. Introduction

### 1.1. Peer Attachment and Prosocial Behavior

Peer attachment denotes the relatively stable emotional bond that individuals form through sustained interactions with peers and reflects the transfer and extension of the attachment system into peer relationships during adolescence (Bowlby, 1969, 1980; Cassidy & Shaver, 2008). Although the concept originates in adolescent attachment theory, it remains highly relevant in emerging adulthood because university students continue to rely on close peers as important sources of emotional security, support, and identity-related validation during the transition to adult roles (Song et al., 2022). In attachment research, peer attachment is typically conceptualized not simply as frequent peer contact or broad social connectedness, but as the perceived quality of close peer relationships, especially in terms of trust, communication, and alienation (Armsden & Greenberg, 1987). With the onset of puberty, peers assume greater importance as sources of emotional support and exert a profound influence on affective development and social adaptation (Rubin et al., 2015; Laible et al., 2000; Tambelli et al., 2012). Attachment theory emphasizes that internal working models shape trust, emotion regulation, and social behavioral strategies, thereby organizing social functioning and interpersonal tendencies (Mikulincer & Shaver, 2019; Fraley & Shaver, 2000). For university students, the quality of peer attachment may be especially important because the college period is marked by increasing autonomy from parents and greater dependence on peers for everyday emotional and interpersonal adjustment (Song et al., 2022).

Prosocial behavior refers to voluntary behavior intended to benefit others, such as helping, sharing, cooperating, and comforting (Malti & Dys, 2018). Among university students, prosocial behavior is an important indicator of positive social adaptation, because it reflects constructive interpersonal engagement and contributes to relationship quality, social integration, and psychosocial adjustment in emerging adulthood (Zhang et al., 2020). Empirical evidence indicates that secure peer attachment is associated with higher levels of prosocial behavior and with lower levels of aggression and other externalizing problems (Rajchert, 2015; Peng et al., 2021; Groh et al., 2014). More directly, evidence from Chinese college students has shown that peer attachment is positively associated with global prosocial behavior, suggesting that secure and supportive peer bonds remain an important relational foundation for prosocial tendencies beyond adolescence (Zhang et al., 2020). By contrast, social exclusion or impaired peer attachment tends to co-occur with reductions in prosocial behavior and increases in externalizing difficulties (Asher & Coie, 1990; Gest et al., 2001; Parker et al., 2015).

Therefore, peer attachment functions not only as a critical individual difference marker for understanding university students’ prosocial tendencies, but also as an important relational basis linking socioemotional experience and concrete social behavior. This interpretation is also broadly consistent with recent social brain perspectives highlighting the close interconnection among social cognition, social bonding, and coordinated human interaction (Oesch, 2024). Taken together, for university students in emerging adulthood, peer attachment captures the emotional quality of close peer bonds, whereas prosocial behavior reflects an important positive behavioral manifestation of social adaptation; this provides a clear theoretical basis for examining their association in the present study (Song et al., 2022; Zhang et al., 2020).

### 1.2. The Mediating Role of Positive Legal Emotion

Perspectives on emotional socialization emphasize that emotional experience is deeply embedded within sociocultural contexts and interpersonal interactions (X. Fu, 2016). In the present study, positive legal emotion is not used in the narrow sense of simple admiration for the justice system alone. Rather, it refers to individuals’ favorable affective responses to legitimate normative order, including trust in, respect for, and emotional identification with rules, authority, and institutional procedures that are perceived as fair, meaningful, and socially valuable (Xu, 2026). This conceptualization is consistent with recent legal socialization scholarship, which defines legal emotions as subjective affective responses elicited by legal stimuli, including procedural interactions, institutional symbols, and the perceived fairness of outcomes (Xu, 2026). Accordingly, although positive legal emotion may be expressed as confidence in law and judicial institutions, it may also extend to broader institutionalized social contexts in which norms are communicated, enacted, and emotionally experienced (Xu, 2026). The present study proposes that positive legal emotion plays a mediating role in the association between peer attachment and prosocial behavior.

First, peer attachment may strengthen individuals’ identification with social institutions and normative systems by providing emotional support, interpersonal trust, and the everyday reinforcement of shared social norms. High-quality peer relationships facilitate the internalization of group values and contribute to favorable evaluations of institutional legitimacy (Laible et al., 2000; Mota & Matos, 2013). For university students in particular, peer relationships are not merely private interpersonal ties; they are also embedded in organizational settings such as classrooms, campuses, and student communities, where rules, authority expectations, and standards of appropriate conduct are continuously communicated and negotiated (Xu, 2026). Through repeated experiences of trust, respect, fairness, and shared norm enforcement within these relational settings, individuals may gradually generalize from concrete interpersonal experiences to more abstract affective support for legitimate institutional and normative order (Xu, 2026). In this way, the psychological transition from the justice system to organizational normativity is achieved not by treating these contexts as identical, but by recognizing that both involve experiences of authority, rule-guided interaction, and judgments of legitimacy (Xu, 2026). Through these relational processes, university students are more likely to develop affective alignment with socially endorsed rules and institutional authority.

Second, positive legal emotion is theoretically and empirically linked to prosocial tendencies. Research on transcendent positive emotions, particularly awe, demonstrates that such emotions expand individuals’ sense of social connectedness and increase identification with broader collective categories, thereby promoting generosity, helping, and cooperative behavior (Bai et al., 2017; Piff et al., 2015; Stellar et al., 2017). In the context of legal socialization, positive legal emotions such as interest, anticipation, trust, and awe have been theorized to strengthen affective identification with legal community and normative order, thereby enhancing the perceived legitimacy and moral worth of rules and institutions (Xu, 2026). When individuals emotionally experience rules and authorities as fair, trustworthy, and worthy of respect, they may become more willing to regulate their behavior in socially approved ways, including helping others, cooperating, and complying with shared norms (Xu, 2026). In a similar vein, positive institutional emotions directed toward law and justice systems can enhance motivation to comply with social norms and heighten concern for others’ welfare, which in turn facilitates altruistic and rule-abiding behavior (Y. N. Fu et al., 2022; Guan et al., 2019).

Taken together, existing theory and empirical evidence suggest that high-quality peer attachment fosters positive legal emotion, which subsequently contributes to stronger prosocial behavioral tendencies. Conceptualizing positive legal emotion as a mediating mechanism allows the present study to connect relational experience with broader institutional normativity: supportive peer attachment may cultivate favorable affective orientations toward legitimate rules and authority, and these affective orientations, in turn, may promote prosocial conduct (Xu, 2026). This formulation also clarifies that the present study does not equate the justice system with all organizational contexts; rather, it proposes that different social contexts may be psychologically linked through shared processes of legitimacy evaluation, emotional identification, and norm internalization (Xu, 2026). Conceptualizing positive legal emotion as a mediating mechanism not only integrates institutional identification into relational and affective accounts of prosocial behavior, but also addresses a notable gap in the literature by foregrounding institutional positive emotion as a distinct psychological pathway through which social relationships shape prosocial conduct.

### 1.3. The Moderating Role of Social Exclusion

For university students, the more normative social condition is social inclusion, often discussed in higher education research as a sense of belonging or feeling that one is a valued part of the university community (Dias-Broens et al., 2024; Gopalan & Brady, 2020). Accordingly, in the present study, social exclusion is not conceptualized as chronic structural marginalization of the kind often examined in highly disadvantaged populations. Rather, it refers to episodic interpersonal experiences of being ignored, rejected, or left out within everyday social interactions, even among individuals who are generally socially integrated (Bernstein et al., 2021; Williams, 2007). Social exclusion refers to experiences of being ignored, rejected, or marginalized in social interactions and constitutes an interpersonal context that severely undermines the need for belonging (Williams, 2007; Du & Xia, 2008). This distinction is important because social inclusion and social exclusion are not mutually exclusive population categories. Even in generally inclusive settings such as universities, students may still encounter exclusionary episodes in peer groups, classrooms, dormitories, or campus activities, and such experiences can meaningfully affect emotion, cognition, and behavior (Bernstein et al., 2021; Gopalan & Brady, 2020).

A large body of research shows that social exclusion elicits negative affect, weakens self-regulation, and alters cognitive processes and behavior (Baumeister et al., 2002; Twenge et al., 2007). At the same time, inclusion-oriented research suggests that belonging and social acceptance are positively associated with students’ well-being, persistence, engagement, and mental health, underscoring that exclusion should be understood against the broader backdrop of students’ striving for inclusion and interpersonal connectedness (Dias-Broens et al., 2024; Gopalan & Brady, 2020). With respect to prosocial behavior, some studies report that social exclusion typically suppresses prosocial indicators such as information sharing and charitable giving (Cuadrado et al., 2016; Takhsha et al., 2020), whereas other studies emphasize that exclusion can stimulate compensatory affiliative motives, leading individuals to behave more conformingly or cooperatively in order to restore social connection (Maner et al., 2007; Williams et al., 2000). Recent evidence from college student samples likewise indicates that the effect of social exclusion on prosocial behavior is not uniform: in many cases exclusion predicts lower prosocial behavior, but under some motivational conditions it may also evoke reconnection-oriented responses (Hou et al., 2024).

Taken together, these findings suggest that social exclusion is likely to operate as a contextual boundary condition that moderates the extent to which peer attachment promotes positive legal emotion. More specifically, because university students are typically embedded in a broader context of social inclusion, exclusion may be best understood here as a situational disruption of belonging rather than as a fixed social status. Under such conditions, exclusion experiences may weaken the emotional security and normative affirmation ordinarily derived from peer attachment, thereby constraining the extent to which peer attachment fosters positive legal emotion and, indirectly, prosocial behavior. In other words, social exclusion may condition the indirect pathway from peer attachment through positive legal emotion to prosocial behavior, a pattern that can be formally tested within a moderated mediation model.

### 1.4. Current Study and Hypotheses

We tested a moderated mediation model in which peer attachment promotes university students’ prosocial behavior via positive legal emotion, and social exclusion moderates the path from peer attachment to positive legal emotion. Framed by attachment theory and the emotional socialization perspective, and drawing on work showing that threats to belonging heighten sensitivity to social and normative cues (Maner et al., 2007; Williams, 2007), we propose that when students experience greater social exclusion, the supportive and normative signals conveyed by peers will more strongly foster institutional affect toward law. Positioning positive legal emotion as the mediating mechanism addresses a gap in the literature that has emphasized legal cynicism and negative legal affect while largely overlooking positive institutional affect (e.g., Piff et al., 2015; X. Fu, 2016; Y. N. Fu et al., 2022). Conditional indirect effects were estimated using Hayes’ PROCESS with 5000 bootstrap resamples, and key covariates were statistically controlled.

**Hypothesis** **1.** *Peer attachment positively predicts prosocial behavior.*

**Hypothesis** **2.** *Positive legal emotion mediates the association between peer attachment and prosocial behavior.*

**Hypothesis** **3.** *Social exclusion moderates the effect of peer attachment on positive legal emotion, such that the positive association is stronger under high (vs. low) social exclusion.*

**Hypothesis** **4.** *Accordingly, the indirect effect of peer attachment on prosocial behavior via positive legal emotion is stronger under high (vs. low) social exclusion (i.e., a conditional indirect effect).*

## 2. Method

### 2.1. Participants

The present study recruited Chinese university students and employed a questionnaire survey. A total of 420 questionnaires were distributed and all were returned. Data screening proceeded according to the following criteria. First, questionnaires with implausibly short completion times were excluded. Based on a pilot study, the average completion time was M = 8.5 min (SD = 1.2). In the formal survey, questionnaires completed in less than 5 min, which is approximately three standard deviations below the mean, were removed to avoid careless responding. Second, internal consistency and response validity checks were applied. Questionnaires exhibiting patterned or random responses identified through reverse-scored items and attention check items were excluded. After screening, 401 valid questionnaires remained, yielding an effective response rate of 95.5%.

The valid sample comprised n = 193 males (48.1%) and n = 208 females (51.9%). The mean age was M = 21.46 years (SD = 1.42). By academic major, n = 247 students were enrolled in science and engineering programs (61.6%), n = 154 in humanities and social sciences programs (38.4%). Parental education levels were distributed as follows. Father’s education: n = 24 primary school or below (6.0%), n = 125 junior middle school (31.2%), n = 153 senior high school or technical secondary school (38.2%), n = 84 junior college or bachelor’s degree (20.9%), and n = 15 above bachelor’s degree (3.7%). Mother’s education: n = 37 primary school or below (9.2%), n = 133 junior middle school (33.2%), n = 146 senior high school or technical secondary school (36.4%), n = 72 junior college or bachelor’s degree (18.0%), and n = 13 above bachelor’s degree (3.2%).

### 2.2. Measures

#### 2.2.1. Positive Legal Emotion Subscale of the College Students’ Legal Emotion Scale

The study employed the Positive Legal Emotion subscale from the College Students’ Legal Emotion Scale developed by Xu et al. (2022). The subscale comprises 11 items, for example, “I believe the people’s courts will adjudicate fairly.” Items are rated on a five-point Likert scale ranging from 1 = “completely disagree” to 5 = “completely agree,” with higher scores indicating greater levels of positive legal emotion. In the current sample, the subscale demonstrated excellent internal consistency, Cronbach’s α = 0.904.

#### 2.2.2. Peer Attachment

Peer attachment was measured using the Peer Attachment Inventory developed by Armsden and Greenberg (1987). The instrument contains 25 items that tap three dimensions: trust, communication, and alienation. Item 5 was reverse-scored. Responses were provided on a five-point Likert scale ranging from 1 (strongly disagree) to 5 (strongly agree). The composite peer attachment score was computed as the sum of the trust and communication subscale scores minus the alienation subscale score, with higher values indicating greater attachment to peers. A representative item is “When something matters to me, I like to ask my friends for their opinions.” In this study, Cronbach’s α for the total instrument and for the trust, communication, and alienation subscales were 0.865, 0.903, 0.915, and 0.637, respectively.

#### 2.2.3. Social Exclusion Experience

Social exclusion experience was evaluated with the eight-item scale developed by Gilman et al. (2013), which comprises two factors: rejection and neglect. The scale was translated into Chinese following standard forward and back translation procedures conducted by one doctoral psychologist and two undergraduate English majors. Items were rated on a seven-point Likert scale from 1 (never) to 7 (always), and higher scores indicate stronger experiences of social exclusion. An example item is “In general, other people ignore me as if I were invisible.” Cronbach’s α for the overall scale and for the rejection and neglect dimensions in the present sample were 0.948, 0.881, and 0.912, respectively.

### 2.3. Procedure and Data Analysis

The study was approved by the Institutional Review Board of Wenzhou University School of Education and conducted after obtaining informed consent from all participants. Data were collected via an online questionnaire distributed to undergraduates at multiple Chinese universities. Procedural safeguards against common method bias included anonymous responding, reverse-scored items, and attention check items. After collection, responses were screened for quality based on completion time, consistency, and attention checks; cases failing these criteria were removed. Participants received a modest, randomly assigned incentive of five to ten Chinese yuan.

Analyses were performed in SPSS 27.0. Descriptive statistics, independent samples *t*-tests, one way analysis of variance, and Pearson correlations were used to summarize the data and examine bivariate relations. Harman’s single factor test indicated 14 factors with eigenvalues greater than one and a first factor accounting for 33.72 percent of variance, below the 40 percent threshold, suggesting no serious common method bias. Conditional process analyses were conducted with Hayes’ PROCESS macro. Controlling for demographic covariates including gender, Model 59 was specified to test whether social exclusion moderates the pathway from peer attachment to positive legal emotion within the model linking peer attachment, positive legal emotion, and prosocial behavior. Indirect and conditional indirect effects were estimated using 5000 bootstrap resamples to obtain bias-corrected confidence intervals.

## 3. Results

### 3.1. Tests of Demographic Differences on Key Variables

Independent samples *t*-tests were conducted to examine gender differences on the primary study variables. Welch’s *t*-tests, which do not assume equal variances, were used for the gender comparisons because Levene’s tests indicated heterogeneity of variance for these variables. Males and females differed only on reported experiences of social exclusion, Welch’s *t*(389.87) = 2.00, *p* = 0.046. There were no significant gender differences for positive legal emotion, Welch’s *t*(363.64) = −1.62, *p* = 0.106; peer attachment total score, Welch’s *t*(390.26) = −1.51, *p* = 0.133; or prosocial behavior, Welch’s *t*(382.79) = −1.45, *p* = 0.148.

Independent samples *t*-tests by academic major found no significant differences on positive legal emotion, *t*(399) = 0.50, *p* = 0.620; social exclusion, *t*(399) = −0.98, *p* = 0.329; peer attachment total score, *t*(399) = 1.55, *p* = 0.123; or prosocial behavior, *t*(399) = 0.24, *p* = 0.813. Levene’s tests for these comparisons were not significant, supporting the assumption of equal variances.

One-way analyses of variance were used to examine age group differences. No significant age effects were observed for prosocial behavior, *F*(3, 397) = 1.11, *p* = 0.346; social exclusion, *F*(3, 397) = 0.28, *p* = 0.838; peer attachment total score, *F*(3, 397) = 1.48, *p* = 0.219; or positive legal emotion, *F*(3, 397) = 0.71, *p* = 0.549.

Analyses of variance by parental education level revealed some differences. Father’s education was associated only with peer attachment total score, *F*(4, 396) = 2.80, *p* = 0.026, and was not significantly related to prosocial behavior, *F*(4, 396) = 1.85, *p* = 0.118; social exclusion, *F*(4, 396) = 0.81, *p* = 0.522; or positive legal emotion, *F*(4, 396) = 0.05, *p* = 0.995. Mother’s education was significantly associated with prosocial behavior, F(4, 396) = 3.84, *p* = 0.004; peer attachment total score, *F*(4, 396) = 6.40, *p* < 0.001; and positive legal emotion, *F*(4, 396) = 3.07, *p* = 0.016. Mother’s education was not significantly related to social exclusion, *F*(4, 396) = 1.31, *p* = 0.264. Levene’s tests for the analyses of variance were not significant, supporting the homogeneity of variance assumption.

On the basis of these subgroup analyses, demographic variables that showed significant associations with the study variables will be included as covariates in subsequent regression and conditional process analyses to improve the precision of model estimates.

### 3.2. Descriptive Statistics and Correlation Analysis for Key Variables

Descriptive statistics and bivariate correlations for the primary study variables are presented in Table 1. Correlation analysis showed that positive legal emotion was negatively associated with social exclusion, *r* = −0.47, *p* < 0.001, and was positively associated with prosocial behavior, *r* = 0.55, *p* < 0.001, and with peer attachment total score, *r* = 0.65, *p* < 0.001. Social exclusion was negatively associated with prosocial behavior, *r* = −0.44, *p* < 0.001, and with peer attachment total score, *r* = −0.57, *p* < 0.001. Peer attachment total score was positively associated with prosocial behavior, *r* = 0.67, *p* < 0.001. All observed correlations were statistically significant and in the hypothesized directions, providing empirical support for the subsequent mediation and moderation analyses. See Table 1 for means, standard deviations, and the full correlation matrix.

### 3.3. Moderated Mediation Analysis

A conditional process analysis was conducted using Hayes’ PROCESS macro (Model 7) to examine whether positive legal emotion mediated the association between peer attachment and prosocial behavior and whether social exclusion moderated the path from peer attachment to positive legal emotion. Gender, father’s education, and mother’s education were entered as covariates in all models. To facilitate interpretation, Table 2 and Table 3 now report the full names of all study variables and provide both unstandardized coefficients (B) and standardized coefficients (β). The unstandardized coefficients indicate the expected change in the dependent variable associated with a one-point increase in the predictor, holding other variables constant, whereas the standardized coefficients indicate the expected change in the dependent variable, in standard deviation units, associated with a one-standard-deviation increase in the predictor.

The total effect of peer attachment on prosocial behavior was significant, *β* = 0.644, *p* < 0.001. This indicates that higher peer attachment was associated with higher prosocial behavior. After inclusion of the mediator, the direct effect of peer attachment on prosocial behavior remained significant, *β* = 0.530, *p* < 0.001, and positive legal emotion was also a significant predictor of prosocial behavior, *β* = 0.209, *p* < 0.001. In other words, as peer attachment increased, prosocial behavior also increased, both directly and indirectly through positive legal emotion.

Moderation analysis indicated that the interaction between peer attachment and social exclusion significantly predicted positive legal emotion, *β* = 0.136, *p* < 0.001. This positive interaction term indicates that the association between peer attachment and positive legal emotion became stronger as social exclusion increased. Thus, the first stage of the mediation pathway was conditional on the level of social exclusion, supporting a moderated mediation model.

Simple slope analyses further clarified this interaction. The positive effect of peer attachment on positive legal emotion increased as social exclusion rose. Specifically, the conditional effects were *β* = 0.416 at low social exclusion, *β* = 0.552 at mean social exclusion, and *β* = 0.688 at high social exclusion, all *ps* < 0.001. Thus, for students experiencing higher levels of social exclusion, increases in peer attachment were associated with larger increases in positive legal emotion. Bootstrapped estimates showed that the indirect effect of peer attachment on prosocial behavior via positive legal emotion was significant at all three levels of social exclusion. The indirect effect was 0.087, 95% *CI* [0.039, 0.152], at low social exclusion; 0.115, 95% *CI* [0.052, 0.194], at mean social exclusion; and 0.144, 95% *CI* [0.063, 0.248], at high social exclusion. These results indicate that the mediating role of positive legal emotion became progressively stronger as social exclusion increased.

The index of moderated mediation was significant, index = 0.028, 95% *CI* [0.005, 0.064], indicating that the indirect effect of peer attachment on prosocial behavior through positive legal emotion became stronger as social exclusion increased. Taken together, these findings suggest that a higher level of peer attachment predicted a greater increase in positive legal emotion, which in turn predicted a greater increase in prosocial behavior, and this indirect effect was more pronounced among students with higher levels of social exclusion. See Table 2 and Table 3 for the detailed regression coefficients and bootstrap confidence intervals.

## 4. Discussion

The present study demonstrates that peer attachment has a significant positive effect on university students’ prosocial behavior, that positive legal emotion mediates the association between peer attachment and prosocial behavior, and that social exclusion significantly moderates the first stage of this mediation pathway. Specifically, under conditions of higher social exclusion, the promotive effect of peer attachment on prosocial behavior through positive legal emotion becomes stronger. In other words, all proposed hypotheses H1 through H4 were empirically supported.

Positive legal emotion, as the central mediating variable in this study, was confirmed as a psychological bridge linking peer relationships to prosocial behavior. This finding is highly consistent with the emotional socialization framework, which emphasizes that emotions are socially constructed and regulated across development through repeated interpersonal interactions and broader institutional contexts (Eisenberg et al., 1998; Morris et al., 2007; Gamble et al., 2011). Legal emotion should not be understood as an isolated legal attitude, but rather as an affective outcome of the legal socialization process. Through interactions within families, schools, peer groups, and institutional settings, individuals gradually form cognitive evaluations of law and corresponding emotional responses (Xu et al., 2022; Xu et al., 2024). Within this process, parents, teachers, peers, and even legal authorities function as socialization agents shaping the development of legal emotion (McLean et al., 2019).

In the context of the present study, high-quality peer attachment provides individuals with stable emotional support, a foundation of trust, and salient normative reference points. These relational resources increase the likelihood that peers’ attitudes toward law and legal institutions, such as respect, identification, and reverence, are accepted and internalized as positive legal emotion. In turn, such institutionally oriented positive emotions enhance prosocial tendencies by strengthening motivations grounded in rule compliance, concern for collective interests, and moral considerations. From this perspective, peer relationships influence not only everyday interpersonal behavior, but also behavioral choices related to public norms through an affective and institutionally embedded pathway.

The moderating effect of social exclusion indicates that the association between peer attachment and positive legal emotion varies as a function of exclusion experiences. When individuals encounter higher levels of social exclusion, their need for belonging is strongly activated (Baumeister & Leary, 1995), and their reliance on interpersonal connection and emotional support correspondingly increases (Williams, 2007). Under such conditions, secure peer relationships not only buffer the negative emotional consequences of exclusion (Maner et al., 2007), but may also serve as a critical avenue through which individuals attempt to reestablish social connection (DeWall & Bushman, 2011). As a result, individuals experiencing high social exclusion are more likely to adopt peers’ social norms, value orientations, and institutional attitudes as salient cognitive and affective reference points, thereby strengthening the effect of peer attachment on positive legal emotion.

In addition, social exclusion often elicits relational repair motives (Richman & Leary, 2009). Following exclusion, individuals tend to engage in behaviors that align with group expectations in order to regain social acceptance. Within this process, positive legal emotion may serve an instrumental function. Expressing respect for and identification with law and legal institutions can facilitate the restoration of group trust and social ties. Consequently, by activating belonging needs and repair-oriented motivations, social exclusion amplifies the strength of the pathway through which peer attachment promotes positive legal emotion.

## 5. Conclusions, Limitations, and Future Directions

The present study elucidates a psychological pathway through which peer attachment promotes prosocial behavior via positive legal emotion and demonstrates that social exclusion significantly moderates the effect of peer attachment on positive legal emotion. Specifically, high-quality peer relationships enhance university students’ positive legal emotion, which in turn strengthens their prosocial behavior. At the same time, social exclusion attenuates the positive association between peer attachment and positive legal emotion, thereby indirectly reducing prosocial behavioral tendencies. By focusing on the social formation of legal emotion, these findings clarify the internal psychological mechanisms through which interpersonal relationships influence prosocial behavior during emerging adulthood and provide empirical support for fostering positive legal personality and social adjustment through the improvement of peer relationships and social inclusion.

Several limitations of this study should be noted. First, the cross-sectional survey design precludes definitive causal inferences among peer attachment, positive legal emotion, and prosocial behavior. Future research could employ longitudinal designs or experimental approaches to further examine the dynamic and causal relations among these variables. Second, the reliance on self-report measures may introduce social desirability effects and subjective bias. Subsequent studies could incorporate behavioral tasks or multi-informant assessments to enhance the reliability and validity of findings. Third, the sample was drawn from universities in a specific regional context, which may limit the generalizability of the results. Future research should test the proposed model across diverse cultural and educational settings. Finally, the measurement of social exclusion focused primarily on individuals’ subjective perceptions. Future studies may further distinguish sources of exclusion, such as peers, teachers, or institutional contexts, as well as variations in exclusion intensity, in order to more precisely elucidate the role of social exclusion in the legal socialization process.

## Figures and Tables

**Table 1:** Descriptive Statistics and Correlation Analysis.

Variables	1	2	3	4
1. Positive Legal Emotion	1			
2. Social Exclusion	−0.47 ***	1		
3. Prosocial Behavior	0.55 ***	−0.44 ***	1	
4. Peer Attachment	0.65 ***	−0.57 ***	0.67 ***	1
*M ± SD*	4.47 ± 0.62	20.18 ± 9.93	105.78 ± 18.12	55.86 ± 14.34

*** *p* < 0.001.

**Table 2:** Results of Regression Analysis for the Moderated Mediation Model.

Variables	Model 1 (Mediating Variable: PLE)			Model 2 (Dependent Variable: PB)		
	*β*	*SE*	*t*	*β*	*SE*	*t*
Constant	1.127	0.665	1.695	−1.814	0.655	−2.769 **
PA (X)	0.552	0.047	11.805 ***	0.530	0.049	10.803 ***
PLE				0.209	0.049	4.303 ***
SE (W)	−0.108	0.047	−2.310 *			
X × W	0.136	0.039	3.500 ***			
Gender	0.038	0.076	0.494	0.061	0.075	0.808
FEL	−0.127	0.062	−2.069 *	0.051	0.061	0.846
MEL	0.059	0.059	1.001	−0.031	0.058	−0.530
R^2^	0.465			0.477		
F	38.073 ***			44.961 ***		

Note.* *p* < 0.05, ** *p* < 0.01,*** *p* < 0.001. PA = peer attachment; PLE = positive legal emotion; SE = social exclusion; PB = prosocial behavior; FEL = father’s education level; MEL = mother’s education level. In Table 2, unstandardized coefficients (B) represent the expected change in the dependent variable for a one-point increase in the predictor, holding other variables constant; standardized coefficients (β) represent the corresponding change in standard deviation units.

**Table 3:** Results of Bootstrap Test for Moderated Mediation Effect.

Social Exclusion	Effect Size	BootSE	Boot 95% CI Lower	Boot 95% CI Upper
Low (−1 SD)	0.087	0.029	0.152	0.039
Medium (Mean)	0.115	0.036	0.194	0.052
High (+1 SD)	0.144	0.047	0.248	0.063
Incremental Change	0.028	0.015	0.064	0.005

Note. Conditional indirect effects are presented at low (−1 SD), mean, and high (+1 SD) levels of social exclusion. Larger indirect effects indicate that the mediating role of positive legal emotion becomes stronger as social exclusion increases.

## Data Availability

The raw data supporting the conclusions of this article will be made available by the authors on request.
